# Supplementary material for: Modulation of Distribution and Diffusion through the Lipophilic Membrane with Cyclodextrins Exemplified by a Model Pyridinecarboxamide Derivative
Source: Pharmaceutics. 2023 May 18;15(5):1531. doi: 10.3390/pharmaceutics15051531 (PMC10224004; doi:10.3390/pharmaceutics15051531)
Supplement: Supplementary file 1 [file pharmaceutics-15-01531-s001.zip › pharmaceutics-2383525-supplementary.pdf]

Supplementary Material

Modulation of Distribution and Diffusion through the Lipophilic Membrane with Cyclodextrins Exemplified by a Model Pyridinecarboxamide Derivative

Tatyana Volkova, Olga Simonova and German Perlovich *

G.A. Krestov Institute of Solution Chemistry RAS, 153045 Ivanovo, Russia; vtv@isc-ras.ru (T.V.); ors@isc-ras.ru (O.S.)

* Correspondence: glp@isc-ras.ru; Tel.: +7-(4932)-336990

Table of Contents

Figure S1	Temperature dependences of IPN solubility in 1-octanol and n-hexane (mole fraction scale).	2
Table S1	Molar concentrations (C_2) of IPN, INZ, and iNAM in the organic and aqueous phases of the distribution systems.	3
Table S2	Donor solution concentrations (C), steady penetrate rate - flux (J), and permeability coefficients (P_{app}) of IPN, INZ and iNAM across the PermeaPad barrier at 310.2 K, pH 7.4.	3

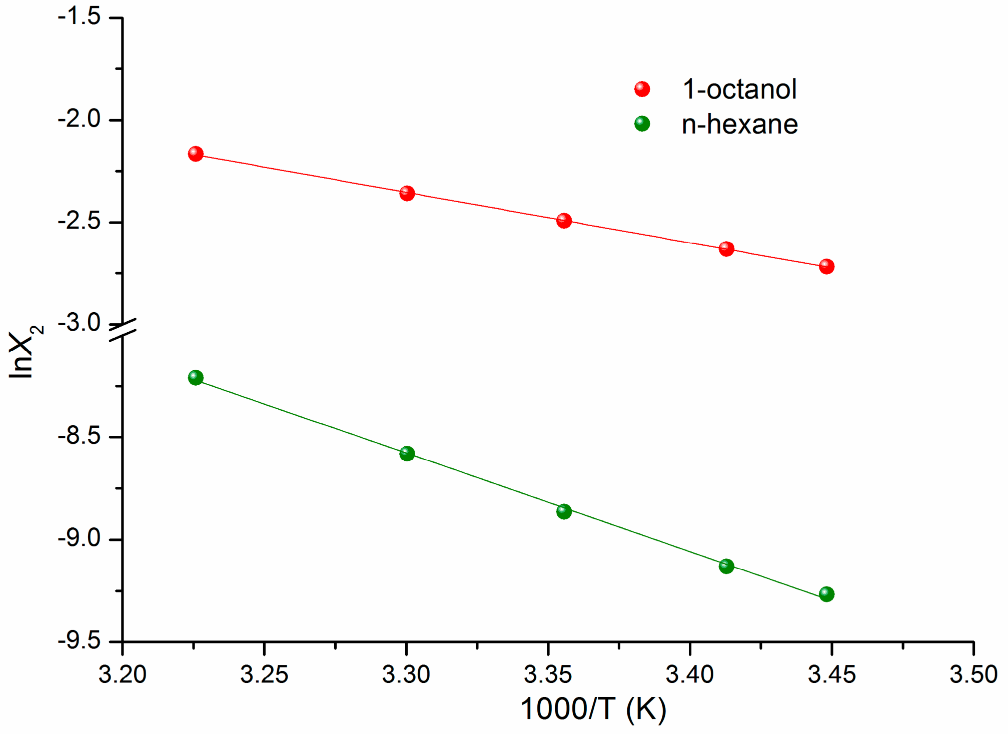

Figure S1. Temperature dependences of IPN solubility in 1-octanol and n-hexane (mole fraction scale).

Table S1. Molar concentrations (C_2) of IPN, INZ, and iNAM in the organic and aqueous phases of the distribution systems.

Compound	$C_2^{oct/buf}\cdot 10^3$	$C_2^{buf/oct}\cdot 10^3$	$C_2^{hex/buf}\cdot 10^5$	$C_2^{buf/hex}\cdot 10^3$
	[a]1-octanol/buffer pH 7.4		[b]n-hexane/buffer pH 7.4	
IPN	4.48	1.69	1.60	6.46
INZ	3.36	5.31	4.67	9.54
iNAM	2.83	6.33	13.71	8.64
IPN/0.0115 M HP-β-CD	4.54	1.88	1.93	6.35
IPN/0.025 M HP-β-CD	4.13	1.93	0.90	6.26
IPN/0.035 M HP-β-CD	4.32	2.25	0.37	6.58
IPN/0.0115 M M-β-CD	4.27	1.67	2.23	6.59
IPN/0.025 M M-β-CD	4.50	1.83	1.43	6.36
IPN/0.035 M M-β-CD	4.05	1.72	1.20	6.52

[a]V(oct):V(buf)= 3:3; [b]V(hex):V(buf)= 6:3 and 10:3 without and with cyclodextrins, respectively.

The standard uncertainties are u(T)=0.5 K. The relative standard uncertainties are $u_r(C_2^{oct/buf})$; $u_r(C_2^{buf/oct})$; $u_r(C_2^{hex/buf})$; and $u_r(C_2^{buf/hex})$=0.04.

Table S2. Donor solution concentrations (C), steady penetrate rate - flux (J), and permeability coefficients (P_{app}) of IPN, INZ and iNAM across the PermePad barrier at 310.2 K, pH 7.4.

System	$C\cdot 10^3$ (M)	$J\cdot 10^4$ ($\mu M\cdot cm^{-2}\cdot s^{-1}$)	$P_{app}\cdot 10^5$ ($cm\cdot s^{-1}$)
IPN	6.13	1.25	2.03±0.05
INZ	-	-	[a]1.44±0.08
iNAM	8.10	0.59	0.73±0.04
IPN/0.0115 M HP-β-CD	5.42	1.02	1.88±0.05
IPN/0.025 M HP-β-CD	5.79	1.05	1.81±0.03
IPN/0.035 M HP-β-CD	6.42	1.14	1.78±0.02
IPN/0.0115 M M-β-CD	6.58	1.64	2.49±0.05
IPN/0.025 M M-β-CD	3.79	1.05	2.77±0.03
IPN/0.035 M M-β-CD	6.16	1.77	2.87±0.04

[a] - taken from [11].
